# Modulating the Properties of Brown Alga Alginate-Based Fibers Using Natural Cross-Linkers for Sustainable Textile and Fashion Applications

**DOI:** 10.1021/acsomega.4c03037

**Published:** 2024-08-21

**Authors:** Ishrat J. Badruddin, Mariana P Silva, Thierry Tonon, Leonardo D Gomez, Sameer S Rahatekar

**Affiliations:** † Composites and Advanced Materials Centre, School of Aerospace, Transport and Manufacturing, Cranfield University, Bedfordshire MK43 0AL, United Kingdom; ‡ Centre for Novel Agricultural Product, Department of Biology, University of York, Wentworth Way, York YO10 5DD, United Kingdom

## Abstract

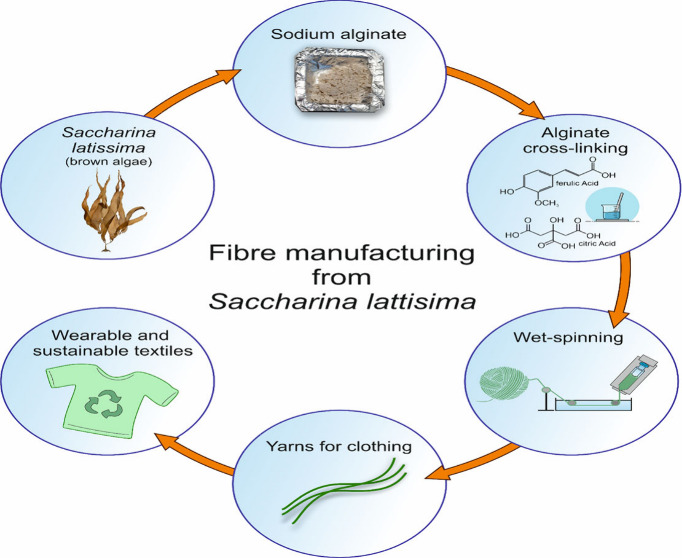

Seaweed-derived alginate shows promise in the textile industry as a sustainable alternative to synthetic and natural materials. However, challenges arise due to its low mechanical strength. We addressed this limitation by sustainably extracting alginates from European brown algae and employing novel manufacturing methods. Using natural cross-linkers, such as chitosan, ferulic acid, and citric acid, we have successfully modulated the mechanical properties of alginate fibers. Mechanical properties of ferulic acid and citric acid-cross-linked alginate solutions were spinnable, producing fibers with a diameter of 73–75 μm. Ferulic acid cross-linked alginate fibers exhibited stiffness, with a tensile strength of 52.97 MPa and a strain percentage of 20.77, mechanical properties comparable to those of wool, polyester, and rayon. In contrast, citric acid-cross-linked fibers showed partial elasticity, with a tensile strength of 14.35 MPa and a strain percentage of 45.53, comparable to those of nylon. This ability to control the mechanical properties of seaweed-derived fibers represents a significant advancement for their application in sustainable textiles and the fashion industry.

## Introduction

Production and manufacturing of natural and synthetic textile materials are very much dependent on energy, water usage, and chemicals thereby having a large carbon footprint and consequently leading to unsustainable textile materials.^1,2^ In recent years, alginate has become one of the most promising biodegradable polymers to produce textiles because it is cost-competitive, readily available, nontoxic, and it can be easily cross-linked with cations.^3,4^ Alginate is a linear polymer composed of β-d-mannuronic acid (M subunit) and α-l-guluronic acid (G subunit) linked by 1,4-linkages and is one of the most abundant components in brown algae cell walls.^5^ Alginate is arranged in G-G, M-G, and M-M blocks, with the percentage and distribution of M and G blocks impacting the physicochemical properties of alginate.^4^ It is well established that alginate rich in M subunits presents flexible structure and good biocompatibility, while alginate enriched in G subunits has a more rigid molecular structure.^6,7^ Alginate fibers can also be transformed into woven, nonwoven, and knitted materials.^8^ The most common way of producing alginate fibers is by microfluidic spinning or wet spinning to obtain fibers of a few hundred micrometers in diameter. Electrospinning can also be used to manufacture fibers that are nanometers or micrometers in diameter.^9−11^ The addition of natural or synthetic polymers to alginate fibers gives them desirable mechanical properties and enables their use in novel processes such as microencapsulation agents, food analogs, and biomedical applications.^12,13^ Alginates are usually cross-linked using synthetic chemicals such as acrylates, impacting the sustainability and biodegradability of the alginate fibers.^14−17^ We have previously established an environmentally benign alginate extraction method for different European brown algae and showed that alginate extracted from *Laminaria digitata* and *Saccharina latissima* could be successfully spun into functional fibers cross-linked with CaCl_2_.^18^ Following this work, we investigated here the potential of improving the properties of *S. latissima* alginate-derived fibers using natural cross-linkers, namely, chitosan, ferulic acid, and citric acid. These cross-linkers are already known for their potential to modify the mechanical properties of alginate. Cross-linked matrices of alginic acid/chitosan were prepared by Fahmy et al.^19^ Ferulic acid-based sodium alginate edible films were developed by Yerramathi et al. and were shown to be homogeneous, stable, and rigid.^20^ Singh et al. used citric acid as a cross-linker to produce pectin/sodium alginate-based edible films.^21^ However, these cross-linkers have been studied in alginate films but not in fibers, where the properties of composite fibers are expected to differ, since uniaxial alignment usually occurs during fiber spinning and drying. In the present work we have used chitosan, ferulic acid, and citric acid for cross-linking and modifying the mechanical properties of alginate fibers obtained from *Saccharina latissima*.

## Experimental Section

### Sodium Alginate Extraction and Characterization

*Saccharina latissima* was collected in May 2021 at Porthallow, England. The biomass was dried for ∼30 h at 40 °C using a dehumidifier and heater and then ground in a hammer mill.

Alginates were extracted and characterized as previously described.^19^ Briefly, milled seaweed biomass (3% w/v) was mixed with a 4% citric acid solution and shaken (200 rpm) overnight at 30 °C. Citric acid proves to be a suitable alternative to mineral acids for the acidic pretreatment step during extraction.^22^ The biomass was then filtered, washed with distilled water, collected, resuspended in 2% Na_2_CO_3_ solution, and shaken as mentioned above. The soluble fraction was collected by centrifugation for 45 min at 3500 rpm. Alginates were precipitated with absolute ethanol (1:2 v/v), collected through filtration, and freeze-dried overnight. This alginate was referred as SAC hereafter in the text.

Alginate characterization was performed by (1) high-performance anion exchange chromatography (HPAEC; Dionex, U.K.) for assessing the total monosaccharide composition; (2) ^1^H NMR spectroscopy for the determination of mannuronic acid (M) and guluronic acid (G) ratio (M/G), using a JEOL JNM-ECS400A spectrometer (JEOL, Peabody, MA, U.S.A.) at a frequency of 400 MHz for 1 h; and (3) size exclusion chromatography-multiangle laser light scattering (SEC-MALLS) for analysis of the molecular weight.

### Preparation of Solutions

#### 1 Chitosan-Alginate Solution (CH-SOL)

To prepare a solution of CH-alginate, chitosan (CH, 3.21 g) was mixed with 1.6 g of SAC, 1.6 g of citric acid, and 0.8 g of sodium hypophosphite and dissolved in 150 mL of water containing 1.6% v/v glacial acetic acid. Nine mL of glycerol (6% v/v) were added, and the reaction mixture was heated between 75 to 80 °C and stirred for 40 min.^19^

#### 2 Ferulic Acid-Alginate Solution (FA-SOL)

A solution of ferulic acid (FA) was prepared as follows: 834 mg of FA were dissolved in 20 mL of 60% ethanol solution using round-bottom flasks. The solution was initially stirred at room temperature and then continued to stir with a slow increase in the temperature (approximately 5 °C/30 min) for 2 h. To prepare a solution of FA-alginate (final 8% w/v alginate), 1.6 g of SAC was slowly added to 16.3 mL of distilled water containing 1% v/v of glacial acetic acid, and stirred for an hour at 60 °C. Subsequently, 2.5 mL of the FA solution were added to the stirring alginate solution at 50 °C. Finally, 1.2 mL of glycerol were added to the stirring alginate solution.^20^

#### 3 Citric Acid-Alginate Solution (CA-A SOL and CA-B SOL)

Two different solutions of citric acid (CA) and saccharina were prepared separately. Solution CA-A SOL (final 8% w/v alginate) was obtained by dissolving 1.6 g of SAC, 1.6 g of CA, 0.8 g of sodium hypophosphite, and 3 mL of glycerol in 17 mL of water containing 1% v/v of glacial acetic acid. Solution CA-B SOL (final 3% w/v alginate) was prepared by combining 1.6 g of SAC, 1.6 g of CA, 0.8 g of sodium hypophosphite, and 3 mL of glycerol in 50 mL of deionized water containing 1% v/v of glacial acetic acid. Both solutions were then heated between 75 to 80 °C and stirred for 40 min.^21^

Sodium hypophosphite was incorporated into the produced solution as a catalyst for reactions, given its role in catalyzing ester cross-linking reactions and regulating the cross-linking system.^18^ Glycerol (6% v/v) was incorporated into each solution as a plasticizer. This addition was made because glycerol is compatible with alginate and is facilitated by the formation of hydrogen bonds attributed to the hydroxyl functional groups.^20^

### Fourier Transform Infrared Spectroscopy (FT-IR)

FT-IR analyses were performed using a PerkinElmer Frontier, U.K. Spectra were collected in the absorption band range of 800 to 4000 cm^–1^ at room temperature. A total of 32 scans were averaged for each sample at a 4 cm^–1^ resolution. Three spectra were collected for each sample, and the raw data were normalized using linear baseline correction.

### Preparation of Cross-Linked Alginate Solutions for Spinning

Eight % SAC and each prepared solution (CH-SOL, FA-SOL, CA-A SOL, and CA-B SOL) were transferred into a 20 mL syringe, and the loaded syringes were degassed by placing them in a vacuum oven for 6 h to remove any bubbles prior to spinning. For wet-spinning, a 4% (w/v) calcium chloride (CaCl_2_) solution was prepared and transferred to a bath before starting the process.

### Wet Spinning of Produced Alginate Solutions

A lab-built wet spinning apparatus consisting of a syringe pump, a CaCl_2_ bath, and a motor-regulated winding drum was used as described earlier.^18^ Individual alginate solutions of 8% saccharina (8% SAC) and each cross-linked solution CH-SOL, FA-SOL, CA- A SOL, and CA- B SOL were injected into the CaCl_2_ bath using a 0.9 mm diameter needle and extruded at varying velocities depending on the composition of the cross-linked solution, as shown in Table 1. The draw ratio (DR = *V*_2_/*V*_1_) was calculated as the degree of stretching applied to the fluid filament. After spinning, the fibers were air-dried for 24 h and stored in airtight bags for further analysis.

**Table 1:** Extrusion Speed and Winding Speed of Various Alginate Solution

sample	*V*_1_ (mL/h)	*V*_2_ (rpm)
8% SAC	25	55
CH-SOL	25	55
FA-SOL	25	55
CA-A SOL	20	30
CA-B SOL	15	40

### Spinnability

Eight % SAC and each produced solution CH-SOL, FA-SOL, CA-A SOL, and CA-B SOL were extruded in a CaCl_2_ bath, and two main parameters, coagulation ability and spinnability, were assessed during the fiber spinning process, as previously described.^18^

### Diameter Measurements

Eight % SAC and the cross-linked alginate spun fibers were observed under 5× magnification to measure their diameter and to study their morphology. Images were acquired for each 1 cm^2^ sample at three different locations using a Leica (Nikon Eclipse ME600) microscope, and their mean diameter was measured using the LAS Core software, which was further used to calculate the standard deviation in each fiber sample.

### Scanning Electron Microscopy (SEM)

Fibers were coated with 10 nm gold in a vacuum evaporator, and their structures were examined at 20 kV and 1000× magnification with a VEGA3 TESCAN (UK SEM microscope).

### Mechanical Properties

Tensile testing of fibers was performed using a Leica S9D microtest tensile stage controller and Deben microtest software V6.3.4 at 25 °C in a 5 N load cell under a constant deformation rate of 0.2 mm min^–1^. Individual fiber samples were analyzed using a gauge length of 10.2 mm. The individual strand of fiber was pasted onto holding tabs to reduce the clamping impact. The maximum force at break point yielded the ultimate tensile strength and elongation ratio at rupture characterized the strain. Stress–strain curves were obtained using the fiber cross-sectional area measured by microscopy. The Young’s modulus was calculated from the linear slope portion of the stress–strain curve before yield point. All tests were performed with at least three fiber samples for each cross-linked solution. The comparison of mechanical properties of 8% SAC, and cross-linked alginate fibers with other polymers was done using the Cambridge Engineering Selector Software (CES EduPack software, Granta Design Limited, Cambridge, UK, 2009).

## Results and Discussion

### Sodium Alginate Extraction and Characterization

The physical properties of alginate fibers depend on several key factors, including the content in the M and G subunits, and their position in the polysaccharide chains. They are also affected by the molecular weight and the frequency of the GG, MM, and GM blocks in the chain of alginates, with these parameters being influenced by methods of extraction.^21^ Our implemented protocol previously enabled the extraction of 30% (w/w) of sodium alginate from *S. latissima*.^18^ Analysis of monosaccharide content showed that mannuronic acid (M) and guluronic acid (G) were the most abundant constituents of the extract, accounting for 57 and 29 mol %, respectively, with a M/G ratio of 1.89, in agreement with our NMR results.^18^ In addition, alginates from *S. latissima* had a high MW (302 kDa) and a dispersity less than 2 (1.36), as shown in Silva et al.^18^

### Fourier Transformed Infrared Spectroscopy (FT-IR)

**Figure 1:**
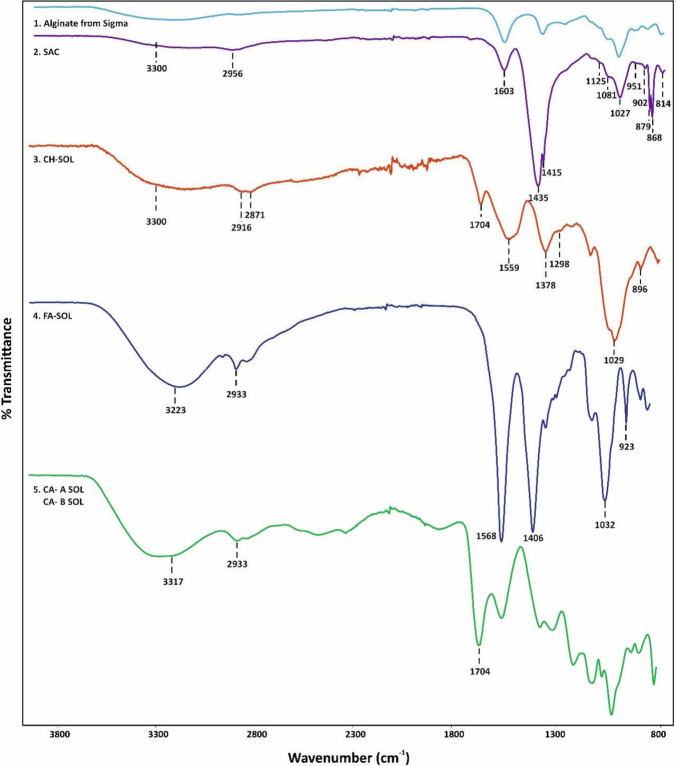
Fourier-transform infrared spectroscopy (FT-IR) spectra of (1) sodium alginate from SIGMA, (2) crude alginate from *S. latissima* (SAC, 8% w/v), (3) chitosan-saccharina cross-linked solution (CH-SOL), and (4) ferulic acid-saccharina cross-linked solution (FA-SOL) and citric acid-saccharina cross-linked solution (CA-A SOL and CA-B SOL).

**Figure 2:**
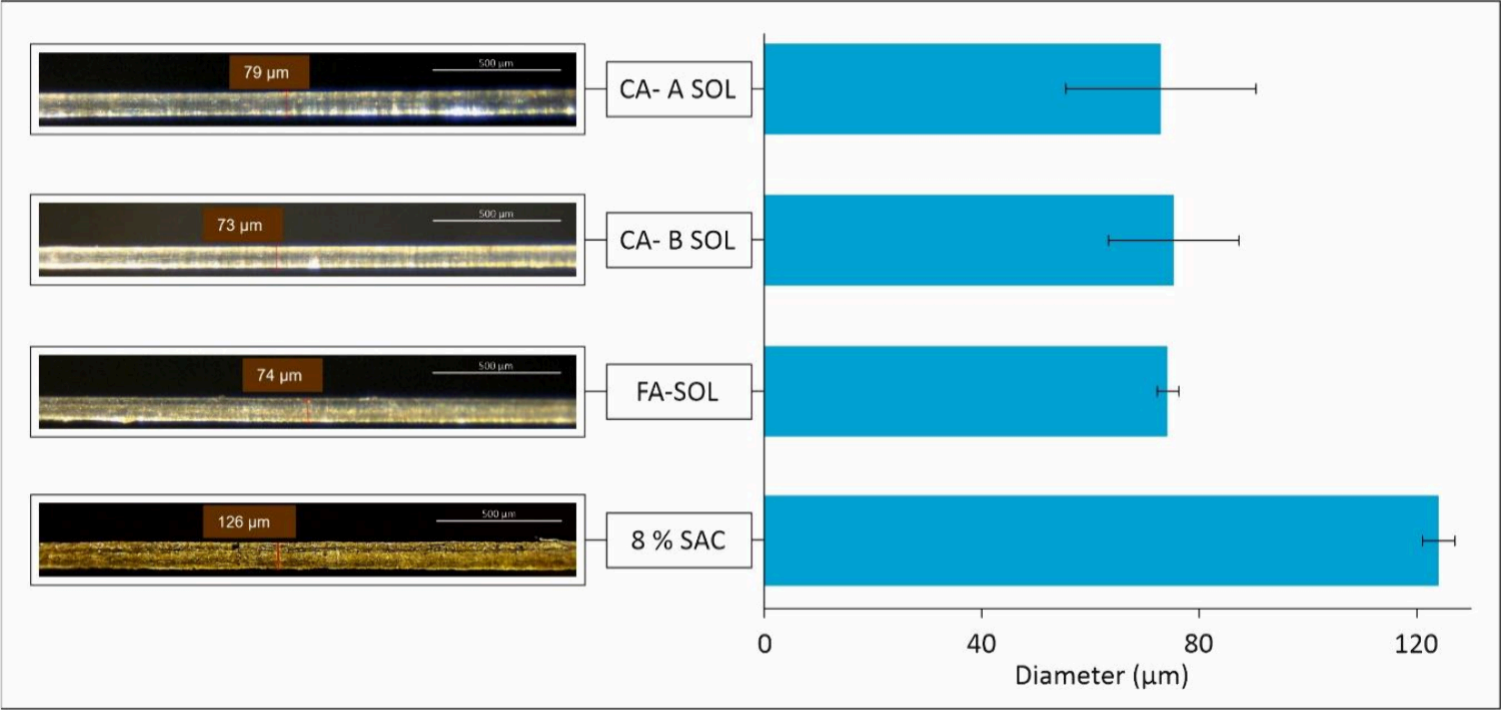
Mean diameter values of fibers and microscopy images of the fibers produced with 8% SAC (126 μm), FA-SOL (74 μm), CA-A SOL (79 μm), and CA-B SOL (73 μm).

The structure of sodium alginate from SIGMA (CAS number: 9005–38–3) used as a control material), SAC (8% w/v), and saccharina after cross-linking was analyzed by FT-IR spectroscopy, and the results are shown in Figure 1. The spectra obtained for sodium alginate from SIGMA and SAC (Figures 1 and 2) exhibit characteristic absorption bands, notably two strong peaks at 1603 cm^–1^ and 1435–1415 cm^–1^, corresponding to the asymmetric and symmetric stretching vibrations of carboxylate groups (COO−), and the contribution of C–OH deformation to the signal at 1435 cm^–1^, as reported previously.^19−21^

Bands observed at 3300 and 2953 cm^–1^ correspond to hydrogen-bonded O–H and C–H stretching vibrations typical of polysaccharides, respectively.^20,21^ Signals at 1125 cm^–1^, 1081 cm^–1^, and 1027 cm^–1^ may be attributed to C–C–H (and O–C–H) deformation, C–O stretching vibrations, and C–O (and C–C) stretching vibrations of pyranose rings, respectively.^7,20,21^ The weak bands at 951, 904.2, and 814.1 cm^–1^ seem related to uronic acids, attributed to C–O stretching vibrations, guluronic asymmetric ring vibration, and mannuronic acid residues.^7^ Additionally, the band at 879 cm^–1^ could be assigned to the Cl–H deformation vibration of the mannuronic acid.^23^

**Figure 3:**
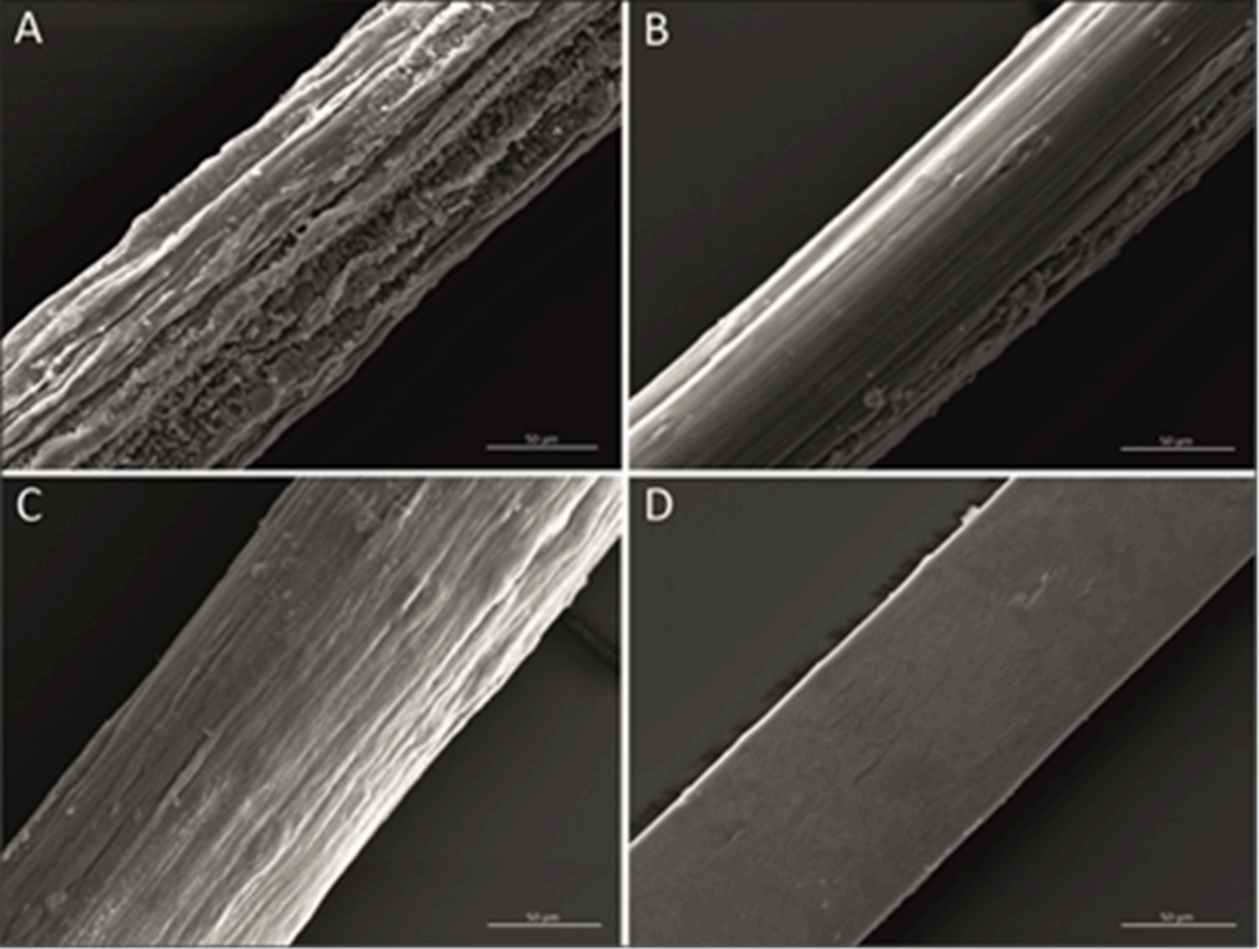
SEM images of fibers produced with 8% SAC (A), FA-SOL (B), CA-A SOL (C), and CA-B SOL (D) solution.

**Figure 4:**
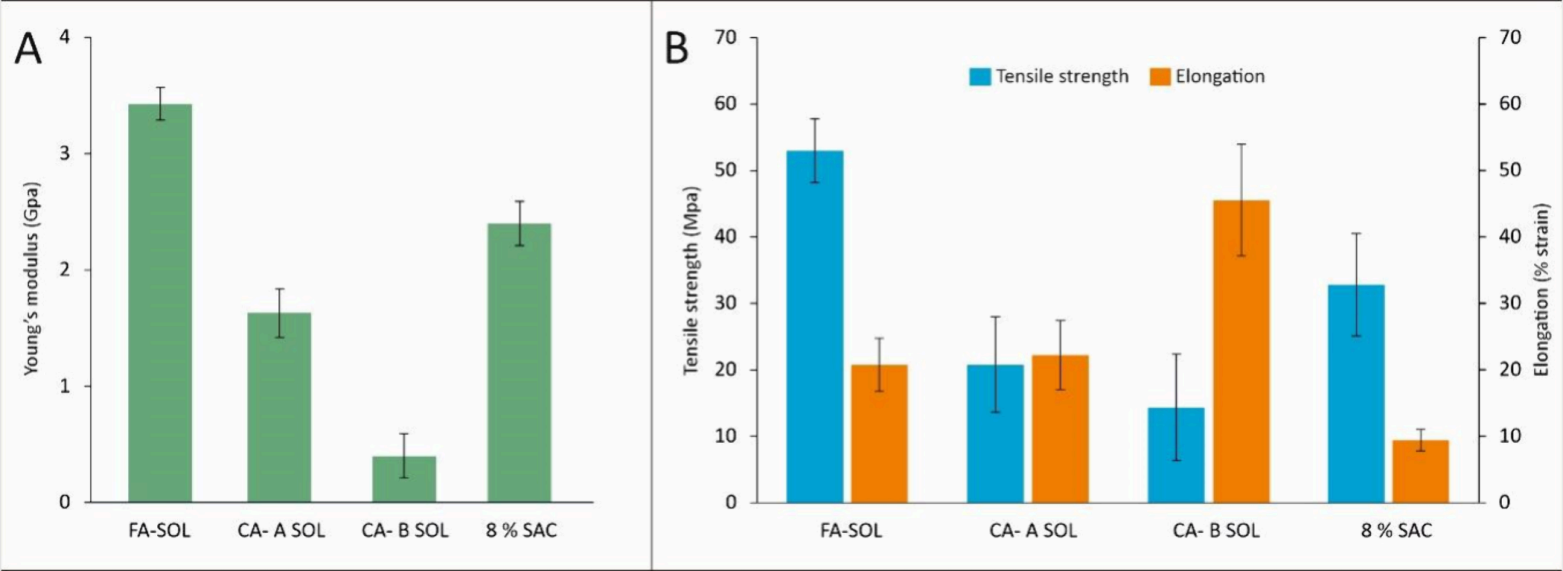
Comparison of Young’s modulus (A), tensile strength, and % strain (B) of the fibers produced with 8% SAC, FA-SOL, CA-A SOL, and CA-B SOL solution.

Figures 1–3 (CH-SOL) present the FT-IR spectra of CH-SOL solution prior to the wet spinning. There were several distinctive bands that show the cross-linking in alginate. The band at 3300 cm^–1^ corresponds to the stretching vibration peaks of the −OH and −NH bonds, while bands at 2916–2871 cm^–1^ are due to the stretching of the C–H of the methyl or methylene group of chitosan and sodium alginate.^19^ Signals at 1559 and 1298 cm^–1^ can be attributed to the bending of the amine of N–H (amide II) and C–N (amide III).^24^ The bands at 1378, 1029, and 896 cm^–1^ usually correspond to the asymmetric stretch of the carboxylate salt of sodium alginate, C–O stretching, and C–O–C stretching, respectively.^19,24^ A band was detected at 1704 cm^–1^, which could be related to an ester carbonyl signal of citric acid, as it was used as a cross-linking agent in CH-SOL.^19^ The FT-IR spectra of FA-SOL solution are shown in Figures 1–4 (FA-SOL). Several characteristic bands were observed. Signal at 923 cm^–1^ in the fingerprint region (not present in Figures 1 and 4 FA-SOL) confirms the presence of O–H bending from the carboxylic group of ferulic acid. The prominent peaks at 3223 and 2933 cm^–1^ correspond to intermolecular O–H and C–H stretching. The band at 1032 cm^–1^ relates to the stretching of C–O. Two strong peaks around 1568 and 1406 cm^–1^ are assigned to asymmetric and symmetric stretching vibrations of carboxyl groups (−COOH) of alginate.^20^ A comparison of the IR spectrum obtained for 8% SAC with the CH-SOL solution spectrum shows that the regions at 3300, 1603, and 1435–1415 cm^–11^ underwent major shifts for 8% SAC (Figure 1). These three bands were shifted to 3223, 1568, and 1406 cm^–1^, and a new peak appears at 923 cm^–1^ in solution FA-SOL, supporting the formation of a chemical bond between ferulic acid and alginate from 8% saccharina.^20^

The FTIR spectra of citric acid-saccharina alginate cross-linked solution (CA-A SOL, and CA-B SOL; Figure 1) shows a broad band at 3317 and 2933 cm^–1^ for the intermolecular O–H and C–H stretching vibration, and a prominent peak at 1704 cm^–1^ associated with an ester carbonyl signal of citric acid that confirms the cross-linking reaction.^21^

### Assessment of the Spinnability of Cross-Linked Alginate Solutions

Table 2 shows the coagulation and spinnability of 8% SAC, CH-SOL, FA-SOL, CA-A SOL, and CA-B SOL solutions. Alginates from 8% SAC, FA-SOL, CA-A SOL, and CA-B SOL show coagulation ability and good spinnability (defined as the ability of the alginate solution to be stretched and spun into continuous fibers), whereas alginate from CH-SOL exhibits no coagulation ability and poor spinnability. Coagulation is the consolidation of the extruded alginate solution into a fiber as it enters the coagulation bath, and spinnability is the ability to continuously stretch the alginate solution into fibers during the fiber winding process. Solutions that consolidated in the coagulation bath to produce fibers were considered to have good spinnability. If incomplete coagulation occurred or the solution did not produce fibers in the coagulation bath and broke due to the stretching action, the solution was considered to have poor spinnability. Solutions of 8% SAC, FA-SOL, CA-A SOL, and CA-B SOL coagulated to produce fibers and were considered as having good spinnability.

**Table 2:** Coagulation and Spinnability of 8% SAC, FA-SOL, CA-A SOL, and CA-B SOL Solution

type of alginate cross-linking	coagulation	spinnability
8% SAC	√	good
CH-SOL	×	poor
FA-SOL	√	good
CA-A SOL	√	good
CA-B SOL	√	good

Based on this observation, the spun fibers obtained with solutions of 8% SAC, FA-SOL, CA-A SOL, and CA-B SOL were air-dried and further characterized.

### Analysis of the Fiber Morphology by Microscopy

The average diameter of the fibers was determined by optical microscopy. The fiber diameter of 8% SAC was 124 μm, whereas the diameter for FA-SOL, CA-A SOL, and CA-B SOL ranges from 73 to 75 μm (Figure 2). Images obtained after spinning 8% SAC and cross-linking solutions FA-SOL, CA-A SOL, and CA-B SOL show that fibers were continuous without any breakage (Figure 2). These results show that adding the cross-linkers to the SAC reduced the average diameter of the fibers significantly. In addition, no signs of larger clumps of alginate or cracks in the fibers were observed. The yellow color observed is due to the intrinsic color of alginate.

### Analysis of Alginate Fibers by Scanning Electron Microscopy

SEM images of the fibers produced by spinning of solutions 8% SAC, FA-SOL, CA-A % SOL, and CA-B SOL are shown in Figure 3. Due to the addition of plasticizer (glycerol) when cross-linking was carried out in FA-SOL, CA-A SOL, and CA-B SOL, the surface of the fibers was smooth, indicating an improved surface finish of the alginate fibers, similar to the alginate fibers obtained in the presence of plasticizer (glycerol).^25^

The addition of glycerol shows a smoother and compact structure due to its plasticizing effect.^26^ The hydroxyl groups of glycerol form hydrogen bonds with alginate, reducing the intermolecular interactions between polymeric chains and disrupting the matrix of alginate. The FA-SOL fibers did not show the presence of grooves and displayed a smooth surface similar to what was observed for fibers obtained by cross-linking sodium alginate and chitosan with ferulic acid.^20,23^ CA-A SOL and CA-B SOL SEM showed smooth fibers similar to the SEM image of the sodium alginate-citric acid wet-spun fibers reported.^20,21,27^

### Mechanical Properties

The tensile properties of fibers produced by wet-spinning of 8% SAC, FA-SOL, CA-A SOL, and CA-B SOL solutions are shown in Figure 4. The addition of ferulic acid in 8% SAC solution showed improved Young’s modulus and tensile strength of the fibers as compared to 8% SAC fibers. Elongation (% strain) of the fiber was improved on the addition of citric acid to the 8% SAC solution. Citric acid is a natural acid capable of stabilizing and cross-linking to the hydroxyl group of polysaccharide through covalent diester linkages, resulting in improved elasticity of the material.^28^ When in excess, the unreacted citric acid reduces the interaction between the polymer chains, allowing them to slide over each other, resulting in reduced tensile strength and increased % strain of the film, as reported by Sharmin et al.^28^

Although FA-SOL, CA-A SOL, and CA-B SOL fibers have similar diameters, their Young’s modulus, tensile strength, and average % strain are significantly different, as shown in Figure 4.

A clear trend is observed where the Young’s modulus and tensile strength of FA-SOL fibers increases as compared to the fibers cross-linked using CA-A SOL and CA-B SOL, as shown in Figure 4A,B. Fibers from CA-B SOL showed significantly higher strain % as compared to FA-SOL, as shown in Figure 4B. Fibers produced from CA-A SOL showed intermediate properties. Glycerol is a small molecule containing three OH groups. Its low molecular weight allows it to penetrate alginate chains and enhance the intermolecular spacing between the polymer chains, known as the “free volume” of the polymer, by creating strong hydrogen bonds with the hydrophilic polymer chains and filling up the empty spaces.^29^ This reduces the intermolecular interactions between polymer chains, making the polymer more flexible and mobile. An increase or excessive amount of glycerol content beyond a certain limit can lower the film strength due to changes in the chemical interactions among the polymer, glycerol, and water.^30^ Addition of low molecular weight glycerol disrupts the inter- and intramolecular hydrogen bonding in the alginate network polymer chains, increasing the free volume, allowing increased movement and increased elongation at break values for the polymer, reducing their tensile strength.^31^

**Figure 5:**
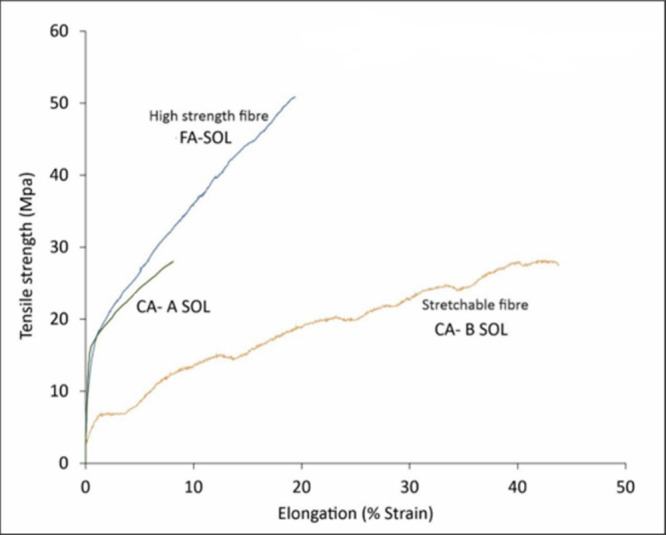
Tensile strength/elongation curve of fibers produced with FA-SOL, CA-A SOL, and CA-B SOL solution.

**Scheme 1:**
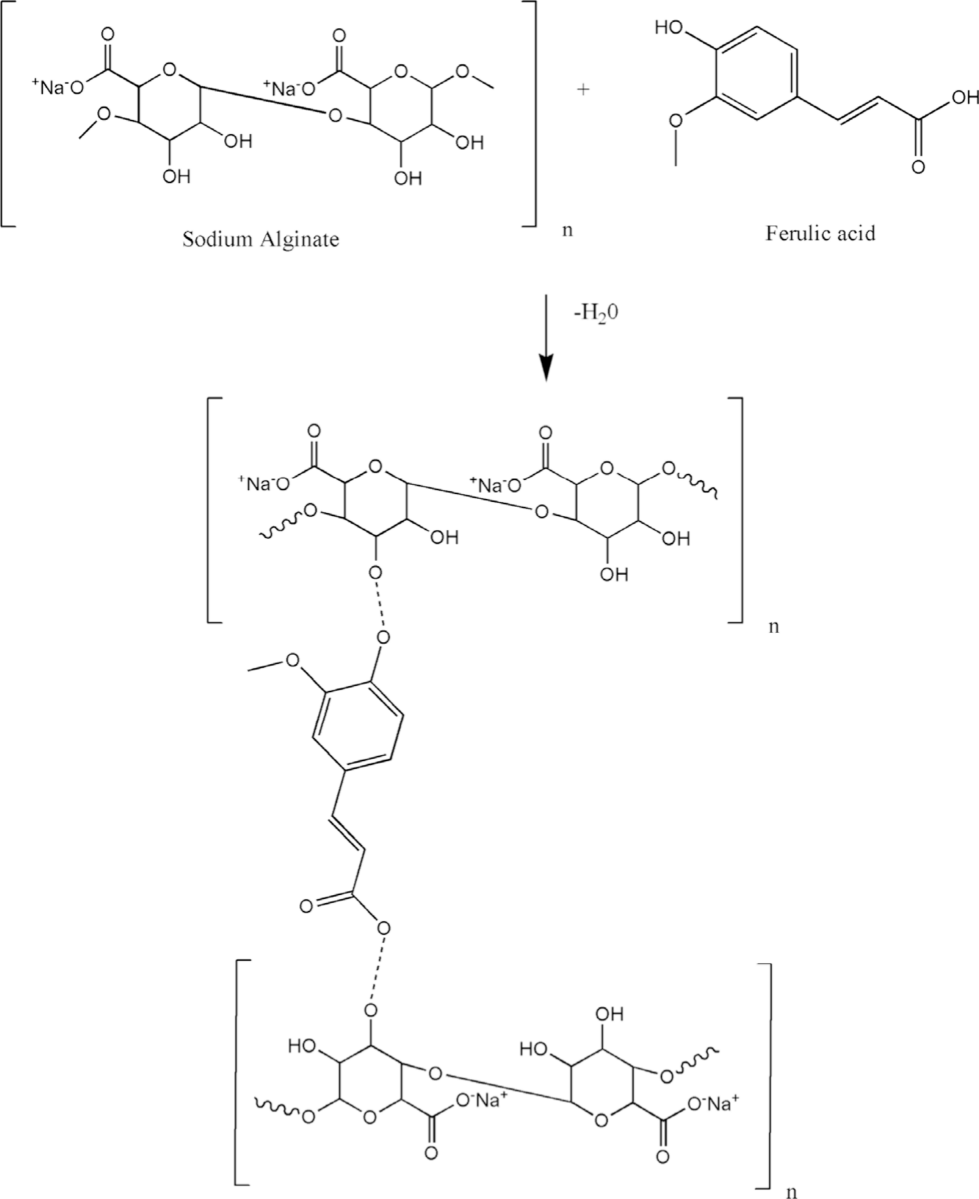
Schematic Representation of the Interaction between Sodium Alginate and Ferulic Acid The dotted line represents the covalent bonding of the OH group of alginate with the carboxyl group of ferulic acid, which results in the removal of a water molecule.

**Scheme 2:**
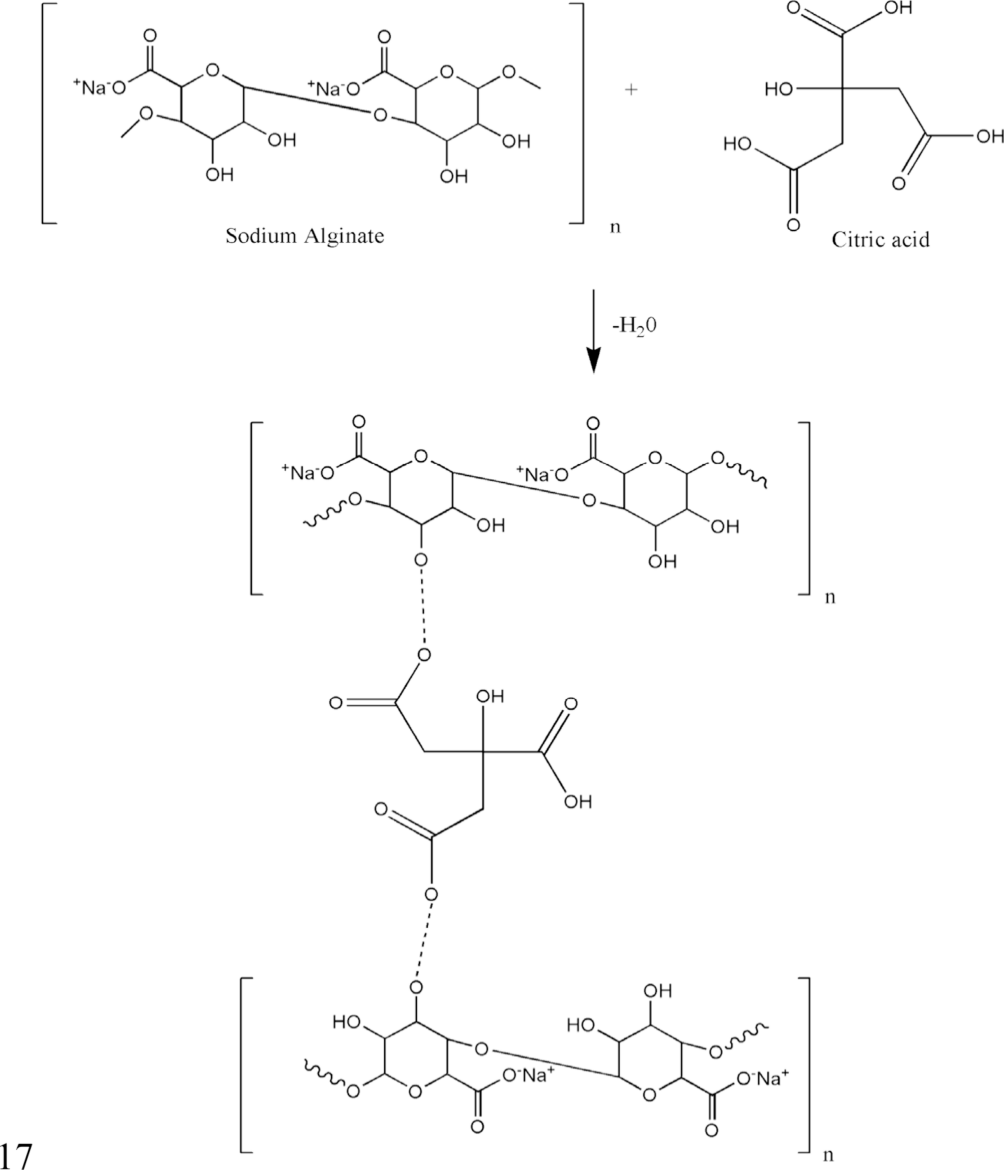
Schematic Representation of the Interaction between Sodium Alginate and Citric Acid The dotted line represents the covalent bonding of the OH group of alginate with the carboxyl group of citric acid, which results in the removal of a water molecule.

When the tensile strength was plotted against the % strain, the fibers showed a linear response until plastic deformation and net failure occurred under different experimental conditions, depending on the properties of the fiber tested. Figure 5 shows the tensile strength/elongation curve of FA-SOL, CA-A SOL, and CA-B SOL fibers. Both ferulic acid and citric acid cross-linkers bond through intermolecular hydrogen bonding with the alginate. The better tensile stress observed for FA-SOL fibers is due to the presence of a ring structure in the ferulic acid (Scheme 1), which enhances the stability of these fibers in comparison with the citric acid cross-linked fiber samples CA-A SOL and CA-B SOL and considering that the citric acid lacks the ring structure (Scheme 2).

These results are in line with a previous demonstration that ferulic acid cross-linking can enhance the tensile strength of the chitosan and sodium alginate films.^23^ A comparison of our FA-SOL fibers with films has been done, as there is limited investigation on producing and cross-linking ferulic acid with alginate to form wet-spun fibers. Our ferulic acid cross-linked alginate fibers have a higher tensile strength (52.97 ± 4.8 MPa) compared to ferulic acid-sodium alginate films (29.67 ± 0.38 MPa), probably because of a better alignment of the OH groups in the fibers caused by the stretching action during the fiber spinning process.^20^ Due to the absence of a ring structure in citric acid, there is an increased movement between the polymer chains in CA-A SOL and CA-B SOL, which results in reduced tensile stress and increased % strain, as previously described.^28^ The hydroxyl group of alginate underwent an esterification reaction by covalent bonding to the carboxyl group of citric acid. Similarly, a plasticizing effect due to high citric acid concentration was obtained by Reddy et al.^32^ Uranga et al. reported a decrease in the tensile strength and an increase in elongation at breaking values for composite film due to a plasticizing effect caused by increasing citric acid content.^33^ Wu et al. reported that, at high concentrations, citric acid will behave as both a cross-linker and a plasticizer in a biopolymer matrix.^34^ This reduction in tensile strength and improved elongation % are attributed to excess residual citric acid behaving as a plasticizer. Therefore, it is evident that, depending on the amount, citric acid may act as a plasticizer and can reduce the interactions between macromolecules, which results in reduced tensile strength and increased strain % values. Aneem et al. reported a 7% elongation and 8.4 MPa tensile modulus for polymannuronate NaAlg-citric-dimethyl sulfoxide fibers; 16.2 and 16.4 MPa tensile modulus for NaAlg-citric-dimethylformamide and NaAlg-citric-tetrahydrofuran wet-spun fibers.^27^ An investigation of the effect of citric acid concentration on poly(vinyl alcohol)/xylan composite films showed that low citric acid percentage with respect to the poly(vinyl alcohol)/xylan film would act as a cross-linker, but high citric acid percentage would act as both cross-linker and plasticizer.^35^ Our present study shows similar results for CA-A SOL, as the strain percentage was lower compared with CA-B SOL fibers and with evident effect of citric acid as both cross-linking agent and plasticizer in the CA-B SOL biopolymer fibers due to extra unreacted citric acid.

**Figure 6:**
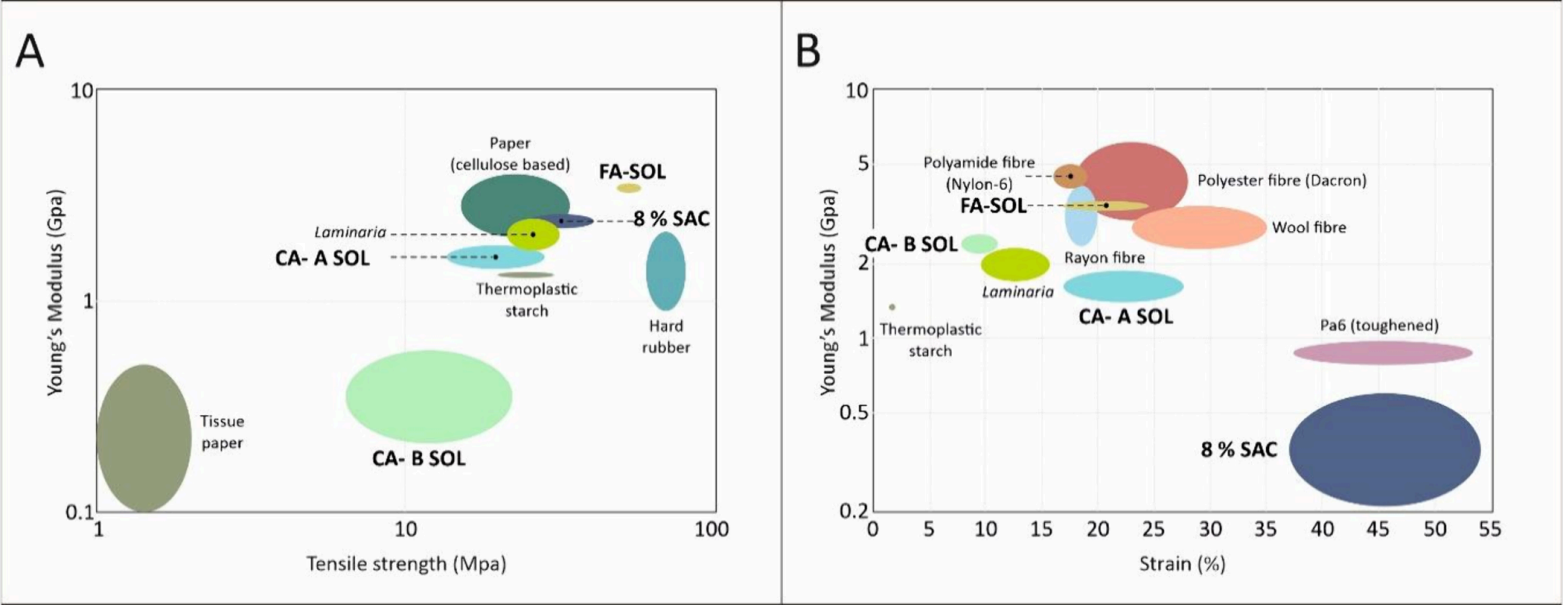
Comparison of Young’s modulus vs tensile strength (A) and Young’s modulus vs % strain (B) of 8% SAC, FA-SOL, CA-A SOL, and CA-B SOL fibers (shown in bold) with previously manufactured *Laminaria* fibers (shown in italic) and other synthetic and natural polymers obtained through Ansys Granta EduPack data.

A better alignment of the OH groups due to the stretching action during fiber formation may provide additional strength to FA-SOL, CA-8% SOL, and CA-B SOL fibers as compared to films.^36^ In addition to having better tensile properties than CA-8% SOL and CA-B SOL fibers, FA-SOL fibers also present higher Young’s modulus when compared with our previous fibers, obtained by spinning of alginate extracted from *Laminaria digitata* and cross-linked using CaCl_2_, and are comparable to other synthetic and natural fibers (Figure 6A,B).^18^

The Young’s modulus of FA-SOL fibers is comparable to other thermoplastic textile materials, such as polyester fibers (Dacron) and Nylon-6 fibers. Dacron is used in a variety of garments, such as shirts, jackets, dresses, and pants, as well as in domestic applications, such as curtains, carpets, and pillows. Although Dacron is a durable, versatile, and lightweight polyester of synthetic origin, it is derived from a polycondensation reaction between monoethylene glycol and pure terephthalic acid, and this manufacturing process results in a negative environmental impact.^37^ Nylon-6 synthetic fibers are produced from caprolactam and are used in the manufacturing of lightweight woven and knitted garments, such as socks, sarees, or furs.^38^ The mechanical properties of FA-SOL fibers are also comparable to natural fibers, such as wool and rayon fibers, used in dresses, linings, shirts, coats, and jackets.^39^ CA-B SOL behaves as a thermoplastic whose % strain is between 37.13 and 53.93, similar to polyamide (Nylon; Type 6, toughened, unreinforced) that has a % strain ranging from 37.2 to 53.5%.^40^ This latter material is used in swimwear and active sportswear.^39^

## Conclusion

This work emphasizes the potential of European brown algae as a feedstock for sustainable fashion applications, particularly in microfiber manufacturing. There is limited research on producing alginate-based fibers using organic acid pretreatment and their influence on the resultant microstructure of the alginate fibers. Our results demonstrate that the mechanical properties of alginate fibers obtained from *Saccharina latissima* can be modulated to expand the range of applications of these biobased materials. We achieved stiff fibers with good tensile modulus (3.43 GPa) using natural cross-linking agents like ferulic acid and partially elastic fibers (45.53% strain to failure) using citric acid. The tensile strength of FA-SOL (52.97 MPa) microfibers makes them suitable for applications such as jackets and dress fabrics. The high strain percentage of CA- B SOL fibers (45.53%) implies their potential use in partially elastic fabric materials like jeans, skirts, waistbands, and sportswear. Our research work aligns with United Nations Sustainable Development Goals (UN SDGs) 9 (industry, innovation and infrastructure), 12 (responsible consumption and production), and 14 (life below water). Manufacturing microfibers of varying mechanical properties from European brown algae using a sustainable manufacturing approach lays the groundwork toward a secured supply chain, with reduced transportation costs and carbon footprint.

## Data Availability

All underlying data are available in the article itself.

## Dedication

In memory of Professor Simon McQueen-Mason.
